# Cotton boll extraction and single-boll weight estimation based on UAV multispectral imagery

**DOI:** 10.3389/fpls.2026.1772622

**Published:** 2026-02-18

**Authors:** Maoguang Chen, Caixia Yin, Na Su, Tao Lin, Xiuliang Jin, Fengquan Wu, Pingan Jiang, Qiuxiang Tang

**Affiliations:** 1 Engineering Research Centre of Cotton, Ministry of Education/College of Agriculture, Xinjiang Agricultural University, Urumqi, China; 2 Institute of Cash Crops, Xinjiang Academy Sciences, Key Laboratory of Crop Physiology, Ecology and Cultivation in Desert Oasis, Ministry of Agriculture and Rural Affairs, Urumqi, China; 3 Key Laboratory of Crop Physiology and Ecology, Institute of Crop Sciences, Chinese Academy of Agricultural Sciences, Ministry of Agriculture, Beijing, China

**Keywords:** cotton, cotton boll extraction, multispectral imagery, single-boll weight, unmanned aerial vehicle (UAV)

## Abstract

Single-boll weight (SBW) is difficult to estimate after defoliant application because canopy spectra include numerous mixed pixels from lint, soil, and senescent leaves, leading to strong background interference. Here we propose a UAV multispectral workflow that combines object-based boll extraction, spectral feature selection, and machine-learning regression to improve SBW mapping. Data were collected from a two-year drip-irrigated cotton experiment in Xinjiang, China involving four varieties evaluated under five planting densities treatments. Boll extraction was treated as a supervised object-based classification problem, and maximum likelihood, mahalanobis distance, and parallelepiped classifiers were compared. Fifteen vegetation indices were computed from the extracted boll pixels; informative features were identified using Pearson correlation and SHapley Additive exPlanations importance ranking. SBW was then estimated with ridge regression, random forest regression, and neural network regression using an independent validation dataset. Maximum likelihood consistently achieved overall accuracy above 97% with Kappa values above 0.93, outperforming the other classifiers. Indices derived from the red, red-edge, and near-infrared bands, particularly those designed to reduce soil background effects, showed the strongest relationships with SBW and ranked highest in SHAP. The best-performing model, which integrated maximum likelihood-based boll extraction with neural network regression, achieved a coefficient of determination of 0.80 and a root mean square error of 0.31 g on the validation set. Relative errors remained below 15% across different years, varieties, and planting densities. This workflow reduces background interference and enables transferable SBW spatial estimation for breeding evaluation and density and harvest management.

## 1 Introduction

Cotton (*Gossypium hirsutum* L.) is an important cash crop that plays a pivotal role in the textile industry and regional economic development (Chen et al., 2024). Single-boll weight (SBW), as a key component of cotton yield, not only directly determines yield but is also closely associated with harvest timing and fiber quality (Xu et al., 2020; Rehman et al., 2020). Therefore, SBW is widely used as an important trait for variety identification and population-level evaluation. Rapid and accurate monitoring of SBW facilitates the screening of genotypes with superior boll weight (Niu et al., 2023), improves understanding of varietal adaptability to planting density and environmental conditions, and enhances insights into the yield formation process. However, the conventional quadrat harvesting method is labor-intensive, time-consuming, and destructive, which limits its scalability for large breeding populations and practical agricultural production (Zhang et al., 2025).

With the rapid development of remote sensing technologies, unmanned aerial vehicles (UAVs), owing to their flexibility and portability, have provided a new tool for field-scale, high-throughput crop phenotyping (Tong and Zhang, 2025). High-spatial-resolution RGB and multispectral imagery, combined with machine-learning algorithms, has been widely used for the quantitative retrieval of crop traits such as leaf area index, biomass, and yield (Chen et al., 2025; Guo et al., 2021). Previous studies have shown that UAV imagery acquired at appropriate growth stages can support cotton yield or boll number estimation with high accuracy (Chen, 2023). In particular, incorporating multi-temporal data and agronomic variables (Dai et al., 2025; Yeom et al., 2018) can substantially improve the stability and interpretability of estimation models. However, most existing studies have focused on total yield or boll number, whereas SBW as an independent target trait has received limited attention. Moreover, the robustness of SBW estimation models and their applicability across varieties, planting densities, and years remain insufficiently and systematically evaluated.

One of the primary challenges in remotely estimating SBW is background interference. During the optimal period for SBW observation, cotton canopies typically exhibit high porosity, and bright white lint, exposed soil, senescent leaves, and stems collectively generate a large proportion of mixed pixels. When canopy reflectance or vegetation indices are used directly, the boll signal is substantially diluted, thereby reducing sensitivity to SBW and related traits. Explicitly separating cotton bolls from complex backgrounds using image segmentation algorithms may help mitigate this limitation. Early studies mainly relied on RGB imagery with color-space transformations and threshold-based segmentation to extract cotton bolls (Li et al., 2016; Singh et al., 2021). More recently, machine-vision and deep-learning frameworks have been introduced; however, most efforts have remained at the levels of object detection and counting (Liu et al., 2023), with limited work leveraging boll segmentation outputs for quantitative SBW estimation. Meanwhile, the potential of multispectral information—particularly red-edge and near-infrared bands and derived vegetation indices—for SBW retrieval has not yet been fully explored.

Object-based image analysis provides a flexible framework to address the above challenges (Blaschke, 2010). This approach formulates target extraction as a pixel- or object-level classification problem, thereby enabling the joint use of spectral, spatial, and contextual information (Yuan et al., 2020). When object-based image analysis is coupled with machine-learning regression, it can support an integrated “target segmentation–feature extraction–trait estimation” pipeline: high-purity boll pixels are first obtained, boll spectral features are then extracted via masking, and an SBW retrieval model is subsequently developed. However, for UAV multispectral imagery, systematic comparisons of boll extraction performance among different object-based image analysis algorithms remain scarce, and quantitative evaluations linking “segmentation choice–feature sensitivity–SBW estimation performance” are still lacking.

**Figure 1:**
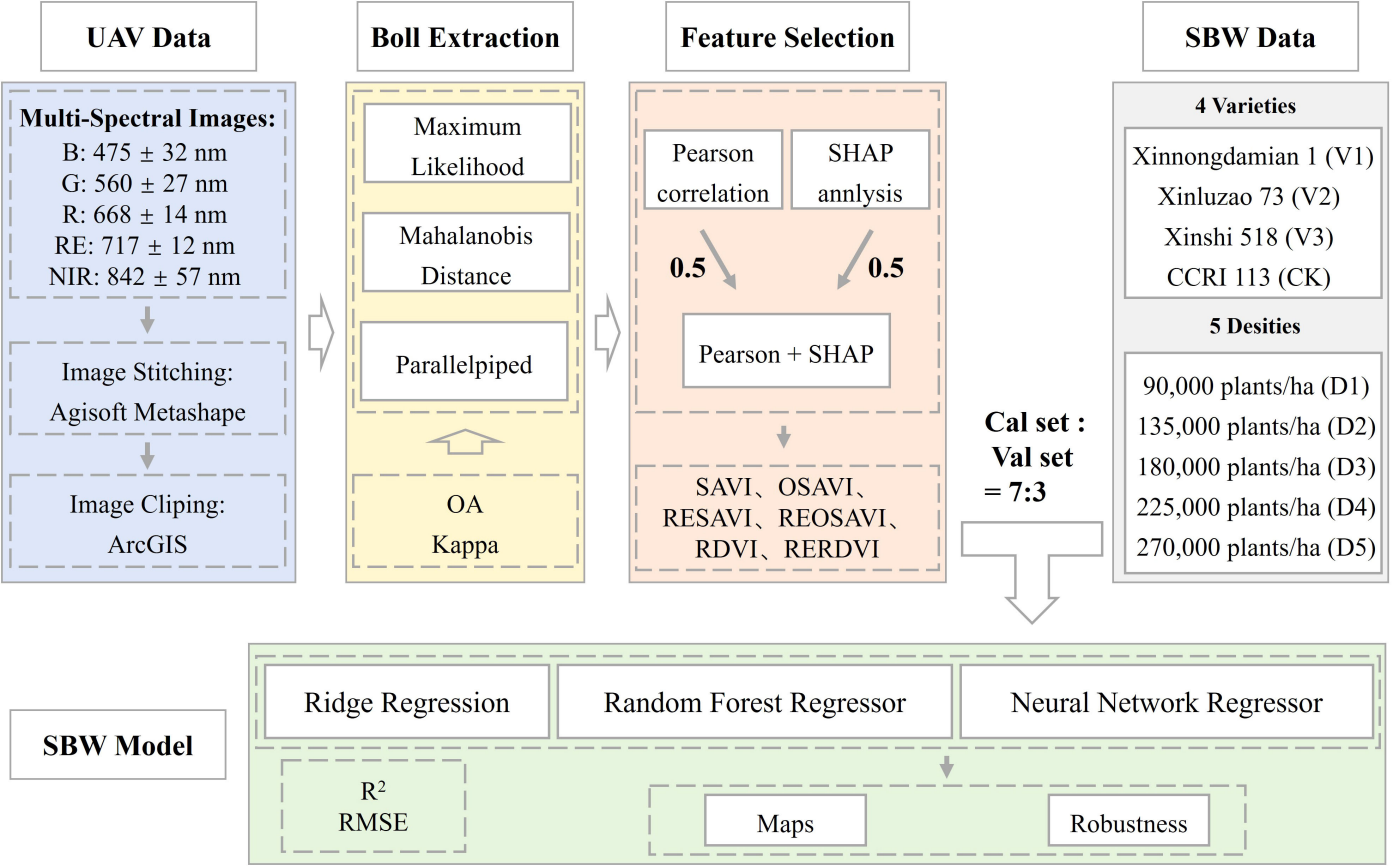
Overall workflow of the proposed method.

Accordingly, we conducted a two-year field experiment in a typical oasis drip-irrigated cotton system in Xinjiang, China, with four varieties and five planting densities. The objectives of this study were to: (i) evaluate the performance of three object-based supervised classification algorithms for cotton boll extraction from UAV multispectral imagery; (ii) identify vegetation indices sensitive to SBW based on pure boll pixels and elucidate the relationship between boll-scale spectral features and SBW; and (iii) develop multiple SBW estimation models using ridge regression, random forest regression, and neural network regression, and assess their robustness across years, varieties, and planting-density scenarios. By explicitly linking boll-scale spectral features with SBW, this study aims to provide a reproducible UAV remote-sensing pipeline for field-scale, high-throughput SBW estimation and to facilitate the integration of SBW and other yield-component traits into UAV-based phenotyping platforms and precision management practices. The overall workflow is shown in **Figure 1**.

## 2 Materials and methods

### 2.1 Study area and experimental design

#### 2.1.1 Study area description

**Figure 2:**
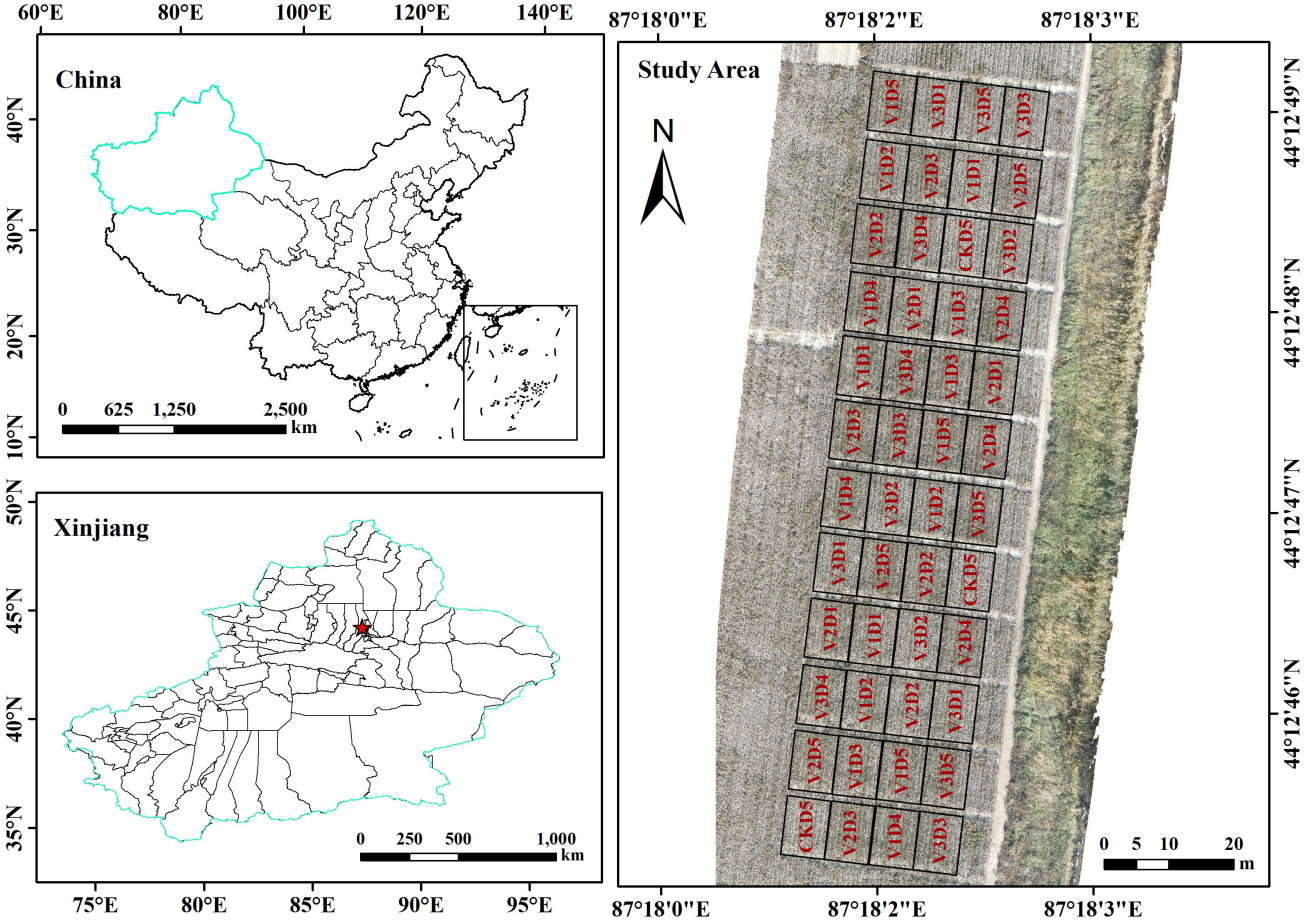
The study area.

As shown in **Figure 2**, the experiment was conducted at Huaxing Farm in Changji City, Changji Hui Autonomous Prefecture, Xinjiang Uygur Autonomous Region, China (44°12′N, 87°18′E), at an elevation of 528 m. The site has a semi-arid continental climate, with an annual mean sunshine duration of 2,700 h, mean annual precipitation of approximately 190 mm, mean annual evaporation of approximately 1,787 mm, and a mean annual air temperature of 6.8 °C. The frost-free period is about 170 days, and the annual accumulated temperature ≥10 °C is 3,450 °C·d; thus, local agricultural production relies entirely on irrigation.

The soil texture at the experimental site is clay loam. Within the plough layer, the mean soil organic matter content was 12.9 g·kg^−1^, total N was 0.67 g·kg^−1^, alkali-hydrolyzable N was 27.49 mg·kg^−1^, available P was 35.65 mg·kg^−1^, available K was 479.5 mg·kg^−1^, bulk density was 1.42 g·cm^−3^, and soil pH was 8.05.

#### 2.1.2 Experimental design

A two-factor randomized complete block design was employed, with planting density and variety as the two factors. Five planting densities were set: 90,000 plants ha^−1^ (D1), 135,000 plants ha^−1^ (D2), 180,000 plants ha^−1^ (D3), 225,000 plants ha^−1^ (D4), and 270,000 plants ha^−1^ (D5). The corresponding within-row plant spacing for the five densities was 29.2, 19.5, 14.6, 11.7, and 9.7 cm, respectively. Four cotton varieties were included: Xinnongdamian 1 (V1), Xinluzao 73 (V2), Xinshi 518 (V3), and CCRI 113 (CK). The CK variety was tested only at D5, resulting in a total of 16 treatments. Each treatment had three replicates, yielding 48 plots in total. Each plot measured 6.9 × 9 m². The cropping pattern followed the local “one film–three drip lines–six rows” system, with a row-spacing configuration of [(10 + 66 + 10 + 66 + 10) + 66] cm. In 2023, sowing and harvest dates were 28 April and 8 October, respectively; in 2024, sowing and harvest dates were 30 April and 26 September, respectively. Other field management practices followed local standards.

### 2.2 Data acquisition

#### 2.2.1 Multispectral UAV image acquisition

**Figure 3:**
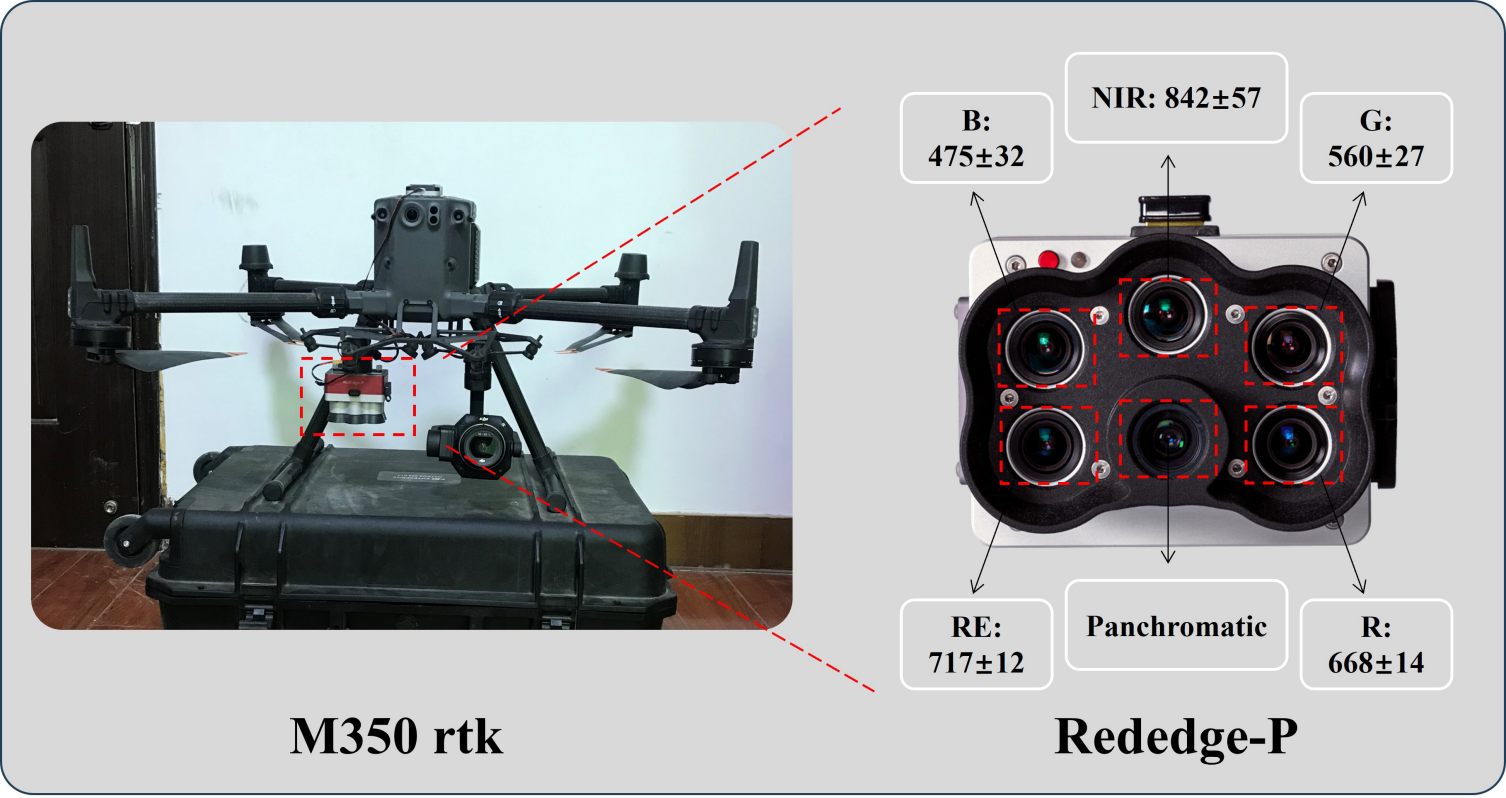
M350 rtk UAV platform and RedEdge-P sensing.

UAV imagery was acquired on the two harvest dates (8 October 2023 and 26 September 2024) using a DJI Matrice 350 rtk platform equipped with a RedEdge-P multispectral sensor (MicaSense, USA) (**Figure 3**). The sensor measures 8.9 × 7.0 × 6.7 cm and weighs 363 g, comprising five discrete spectral bands (blue, green, red, red-edge, and near-infrared) channel. The center wavelengths and bandwidths of the five bands are 475 nm (32 nm), 560 nm (27 nm), 668 nm (14 nm), 717 nm (12 nm), and 842 nm (57 nm), respectively. The image resolution is 1,456 × 1,088 pixels, with a 50° horizontal field of view (HFOV) and a 38° vertical field of view (VFOV).

Image acquisition was conducted under clear, cloud-free, and windless conditions around solar noon (13:00-15:00). Prior to each flight, the UAV was magnetically calibrated. Takeoff and landing were performed at the same location on an open and flat area. A reflectance calibration panel was placed in the cotton field to facilitate radiometric calibration and to identify the field location in the imagery.

The flight altitude was 30 m and the flight speed was 2 m·s^−1^, with both forward and side overlaps set to 85%. Images were captured at equal intervals along the flight path.

#### 2.2.2 SBW data collection

To ensure that the sampled SBW was representative of the mean SBW within each sampling point, a stratified random sampling strategy was adopted. Following the conventional classification of within-plant boll distribution in cotton physiology, bolls were stratified by the vertical position of fruiting branches (i.e., sympodial branch “nodes/tiers”) into three layers: the lower layer (1st-3rd fruiting branches), middle layer (4th-6th fruiting branches), and upper layer (7th-9th fruiting branches or the top fruiting branches). Within each sampling point, 30 normally opened bolls without visible pest or disease damage were randomly collected from each layer. Thus, 90 bolls were collected per sampling point, air-dried to constant weight, and the mean SBW was calculated (g).

**Figure 4:**
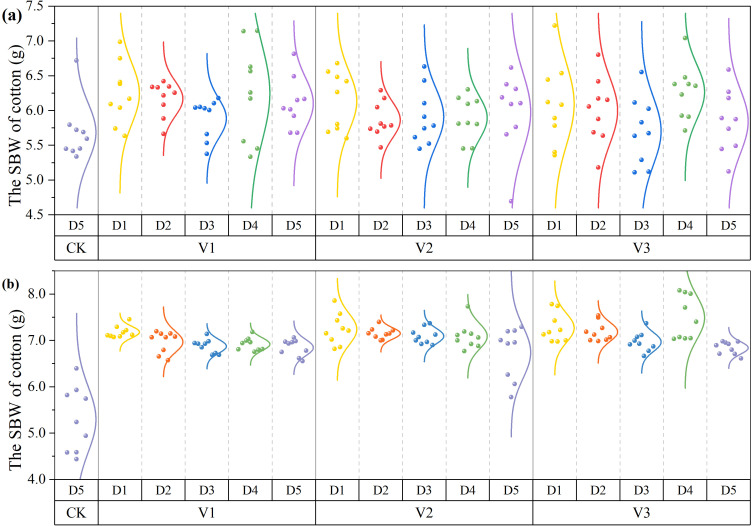
Measured SBW of cotton: **(a)** 2023 and **(b)** 2024.

Each plot was divided into three sampling points according to the number of plastic mulch films, resulting in 144 samples from 48 plots per year and 288 samples over the two years. The distribution of measured SBW is shown in **Figure 4**, and SBW exhibited a decreasing trend with increasing planting density.

### 2.3 Feature construction

#### 2.3.1 Remote-sensing image preprocessing

Image mosaicking was performed in Agisoft Metashape Professional (v1.8.0; Agisoft LLC, St. Petersburg, Russia). First, the acquired multispectral images and the calibration panel images were imported. Using the Multi-camera system workflow, the calibration images were moved to a separate folder. Reflectance calibration was then conducted using the calibration panel, with band-specific reflectance coefficients of 0.512, 0.514, 0.514, 0.513, and 0.511. During “Align Photos”, the key point limit was set to 40,000, followed by “Optimize alignment” and “Build dense point cloud”. A digital elevation model was generated from the dense point cloud, and orthomosaics were subsequently produced.

Following the MicaSense-recommended reflectance calibration procedure in Metashape, the orthomosaic outputs were saved as 16-bit integer values with a scale factor of 32,768. During orthomosaic export, a raster transformation was applied, and each band was normalized using the raster calculator. Ultimately, a five-band reflectance raster was generated.

#### 2.3.2 Cotton boll extraction

Cotton bolls were extracted using three object-based supervised classification algorithms. First, the orthomosaics were imported into ENVI (v5.6; Exelis VIS, Boulder, CO, USA). Three land-cover classes—soil, cotton stems/leaves, and cotton lint—were labeled using regions of interest. For each class, 20 regions of interests were manually delineated and evenly distributed across the experimental plots. The imagery was then classified using three supervised classification tools implemented in ENVI. A 3 × 3 confusion matrix was obtained for each method (**Table 1**), from which classification accuracy metrics were calculated. Subsequently, the cotton lint class in each classified map was converted to a vector layer, which was used as a mask to extract boll pixels from the multispectral imagery, thereby achieving cotton boll extraction. The three object-based algorithms were as follows:

**Table 1:** Confusion matrices for the classification models.

True value	Soil	Predicted value		
		Cotton stems and leaves	Cotton lint	Total
Soil	A_11_	A_12_	A_13_	∑A_1j_
Cotton stems and leaves	A_21_	A_22_	A_23_	∑A_2j_
Cotton lint	A_31_	A_32_	A_33_	∑A_3j_
Total	∑A_i1_	∑A_i2_	∑A_i3_	N

A_11_, A_22_, and A_33_ denote the numbers of correctly classified pixels for each class.

##### 2.3.2.1 Maximum likelihood

Maximum likelihood (Chen et al., 2025), a supervised classification method based on probabilistic decision functions and Bayes’ rule. It estimates the mean and variance of each class from regions of interest pixel values, constructs class-specific discriminant functions, and assigns each pixel to the class with the highest posterior probability. This method is simple and efficient but assumes that the data follow a normal distribution.

##### 2.3.2.2 Mahalanobis distance

Mahalanobis distance (Zeng et al., 2021), an object-based classification approach based on the covariance matrix of multiple features, which effectively quantifies relative distances among data points. By accounting for correlations among features, it is suitable for complex real-world scenarios.

##### 2.3.2.3 Parallelepiped

Parallelepiped (Wilczkowiak et al., 2005) classifier operated in the 5-dimensional feature space of our multispectral imagery. For each of the three target classes, a unique decision region was defined. This was done by calculating the mean and standard deviation of training pixels in all 5 bands. A pixel was assigned to a class only if its value in every band fell within the class-specific range (mean ± a scaled standard deviation). Pixels not meeting any class criteria remained unclassified.

In this study, overall accuracy and the Kappa coefficient were used to evaluate the performance of the segmentation/classification models, and were calculated as shown in Equations 1 and 2 (Huang et al., 2025).

(1) $$\text{Overall Accuracy}=\frac{\sum_{\text{i}}^{\text{k}}{\text{TP}}_{\text{i}}}{\text{N}}$$

(2) $$\text{Kappa}=\frac{{\text{p}}_{0}-{\text{p}}_{\text{e}}}{1-{\text{p}}_{\text{e}}}$$

TP_i_ denotes the number of pixels correctly predicted as class i, and N denotes the total number of pixels. p_0_ represents the observed agreement (i.e., overall accuracy), and p_e_ represents the expected agreement by chance.

#### 2.3.3 Vegetation index calculation

The extracted boll imagery was imported into ArcGIS (v10.6; Esri Inc., Redlands, USA). Regions of interest corresponding to each sampling point were then delineated, and the mean reflectance within each the regions of interest was extracted as the spectral information of that sample. Because no standard vegetation indices have been specifically developed for estimating cotton SBW, we constructed a comprehensive spectral feature pool. Specifically, 15 commonly used vegetation indices, representing six major spectral response categories (difference, normalized difference, soil-adjusted, triangular, non-linear, and other indices), were calculated (**Table 2**). These indices are sensitive to different attributes, including vegetation greenness, soil background effects, and canopy structure, thereby providing a solid basis for exploring the spectral responses associated with SBW.

**Table 2:** Formulas of vegetation indices used in this study.

Index categories	Vegetation indexes	Abbreviation	Formulas	References
Difference	Difference Vegetation Index	DVI	nir−r	(Chen et al., 2025)
Difference	RedEdge-Difference Vegetation Index	REDVI	nir−re	(Killeen et al., 2024)
Normalized Difference	Normalized Difference Vegetation Index	NDVI	((nir−r))−((nir+r))	(Chen et al., 2025)
Triangular	RedEdge Triangle Vegetation Index	RTVI	100 * (nir−re)−10*(nir−g)	(Kushal et al., 2024)
Soil-Adjusted	Enhanced Vegetation Index	EVI	((2.5 * (nir−r)))−((nir+6 * r−7.5 * b+1))	(Chen et al., 2025)
Soil-Adjusted	Soil Adjusted Vegetation Index	SAVI	(1+L) * ((nir−r))−((nir+r+L))	(Kushal et al., 2024)
Soil-Adjusted	RedEdge Soil-Adjusted Vegetation Index	RESAVI	$\left(1+L)\;*\;(\frac{\text{nir}-\text{r}}{\text{nir}+\text{r}+0.5})\;*\;((\text{nir}-\text{re})/(\text{nir}+\text{re})\right)$	(Zhang et al., 2022)
Soil-Adjusted	Optimized Soil-Adjusted Vegetation Index	OSAVI	(1+Y) * ((nir−r))−((nir+r+Y))	(Dai et al., 2025)
Soil-Adjusted	RedEdge-Optimized Soil-Adjusted Vegetation Index	REOSAVI	(1+Y) * ((nir−re))−((nir+re+Y))	(Cao et al., 2013)
Normalized Difference	Renormalized Difference Vegetation Index	RDVI	$\left(nir-r)/(\sqrt[{2}]{\left(nir+r\right)}\right)$	(Lu et al., 2021)
Normalized Difference	RedEdge Renormalized Difference Vegetation Index	RERDVI	$\left(nir-re)/(\sqrt[{2}]{\left(nir+re\right)}\right)$	(Cao et al., 2013)
Non-Linear	Non-Linear Index	NLI	((nir)^2−r)−((nir)^2+r))	(Dai et al., 2025
Non-Linear	Non-Linear Vegetation Index	NLVI	((re)^2−r)−((re)^2+r))	(Li et al., 2013)
Triangular	Transformed Difference Vegetation Index	TVI	60 * (nir−g)−100 * (r−g)	(Kushal et al., 2024)
Other	Modified Simple Ration Index	MSR	$\left(nir/r-1)/(\sqrt[{2}]{(\frac{nir}{r})+1}\right)$	(Killeen et al., 2024)

nir, r, g, b, and re represent reflectance in the near-infrared, red, green, blue, and red-edge bands, respectively. L and Y are the soil adjustment factor (typically 0.5 and 0.16).

#### 2.3.4 Feature optimization and selection

Although using the full set of indices might yield marginally similar predictive accuracy, the feature selection process is essential to develop a parsimonious, stable, and interpretable model by eliminating redundant signals and reducing the risk of overfitting, which is particularly important for potential operational applications.

To identify the most effective and robust predictors for SBW estimation, we adopted a quantitative feature selection strategy that integrates statistical analysis with model interpretability. First, the Pearson correlation coefficient (|r|) was used to quantify the strength of the linear association between each vegetation index and SBW, while SHapley Additive exPlanations (SHAP) analysis was employed to assess the nonlinear contribution and global importance of each index. Next, features were ranked separately based on the two criteria. The ranking information was then integrated using an equally weighted averaging scheme to prioritize vegetation indices with the strongest predictive power for subsequent model development.

### 2.4 Model development and evaluation

#### 2.4.1 Dataset partitioning

A total of 288 field-measured samples were collected over two years. The dataset was randomly split into a calibration set and a validation set at a ratio of 7:3. The detailed dataset partitioning is provided in **Table 3**.

**Table 3:** Composition of the dataset used for SBW estimation.

Categories	Subcategories	Calibration sets	Validation sets	Total
Densities	D1	38	16	54
	D2	38	16	54
	D3	38	16	54
	D4	38	16	54
	D5	50	22	72
Varieties	V1	63	27	90
	V2	63	27	90
	V3	63	27	90
	CK	13	5	18
Years	2023	101	43	144
	2024	101	43	144

#### 2.4.2 Development of SBW estimation models

To systematically evaluate the capability of different algorithms for SBW estimation, three regression models were adopted: ridge regression, random forest regression, and neural network regression representing linear models, ensemble-learning models, and neural-network approaches, respectively.

##### 2.4.2.1 Ridge regression

Ridge regression is an extension of linear regression that introduces an L2 regularization term into the loss function to alleviate multicollinearity among predictors and improve model generalization (Shahzad et al., 2024). In this study, the regularization parameter *α* was searched over a candidate set of 0.001, 0.01, 0.1, 1, 10, and 100, and the optimal value was selected via cross-validation.

##### 2.4.2.2 Random forest regression

Random forest regression is a representative ensemble-learning method that aggregates multiple decision trees to reduce model variance and effectively capture nonlinear relationships and interactions among features (Gao et al., 2024). The main hyperparameters were tuned as follows: *n_estimators* = 100, 200, 300; *max_depth* = 3, 5, 10; *max_features* = ‘sqrt’, ‘log2’; *min_samples_split* = 2, 4, 6; and *min_samples_leaf* = 1, 2, 4.

##### 2.4.2.3 Neural network regression

Neural network regression can model complex functions through multilayer nonlinear transformations and is suitable for describing nonlinear relationships between spectral features and SBW (Corte et al., 2020). A multilayer feed-forward neural network was used, with the hyperparameter ranges set to: *hidden_size* = 32, 64, 128; *learning_rate* = 0.01, 0.001, 0.0001; *epochs* = 500, 1000; and *batch_size* = 8, 16, 32.

All three models used SBW as the target variable and the vegetation indices selected in Section 2.3.4 as input predictors. Model development was implemented in Python 3.9.13, primarily using open-source libraries such as scikit-learn 1.1.1. Hyperparameter optimization was conducted via grid search combined with five-fold cross-validation, with validation performance used as the selection criterion.

#### 2.4.3 Evaluation of regression model performance

To quantitatively assess the estimation performance of each model, the coefficient of determination (R^2^) and the root mean square error (RMSE) were used as the primary evaluation metrics. Specifically, R^2^ measures the extent to which the model explains the variance in SBW, whereas RMSE reflects the absolute deviation between predicted and observed values. A higher R^2^ (closer to 1) and a lower RMSE indicate better model fit and higher estimation accuracy. The metrics were calculated as shown in Equations 3 and 4 (Jaafar and Sujud, 2025):

(3) $${\text{R}}^{2}=1-\frac{\sum_{\text{i}=1}^{\text{n}}{\left({\text{y}}_{\text{i}}-{\text{x}}_{\text{i}}\right)}^{2}}{\sum_{\text{i}=1}^{\text{n}}{\left({\text{y}}_{\text{i}}-\bar{\text{y}}\right)}^{2}}$$

(4) $$\text{RMSE}=\sqrt{\frac{1}{\text{n}}\sum_{\text{i}=1}^{\text{n}}{\left({\text{y}}_{\text{i}}-{\text{x}}_{\text{i}}\right)}^{2}}$$

where n is the sample size, i denotes the ith sample, xi is the observed value for sample i, y_i_ is the predicted value for sample i, $\bar{\text{y}}$ and is the mean of the predicted values.

## 3 Results

### 3.1 Cotton boll extraction based on object-based algorithms

**Figure 5:**
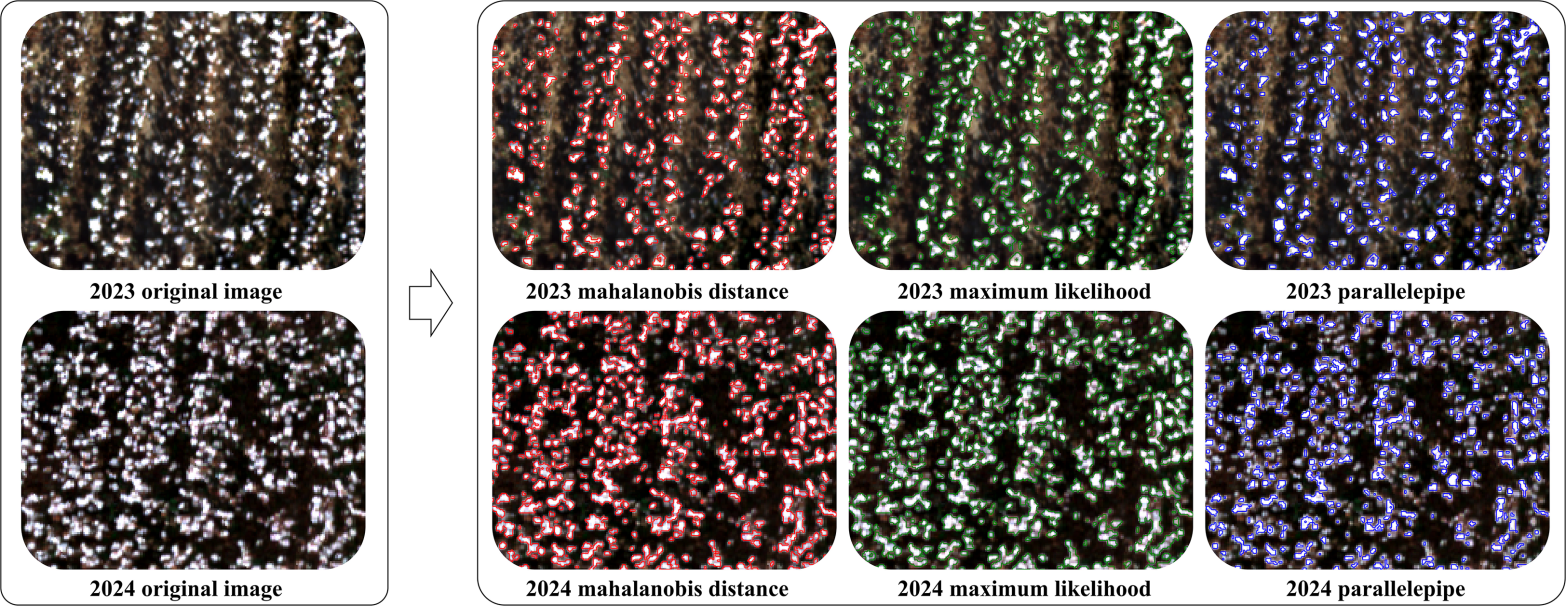
Examples of cotton boll extraction results obtained using object-based algorithms.

In this study, cotton bolls were extracted from UAV imagery acquired in two years using three object-based algorithms, and the accuracy and visual performance of each method are summarized in **Table 4** and **Figure 5**. Both mahalanobis distance and maximum likelihood achieved satisfactory boll extraction, with overall accuracies exceeding 95% and Kappa coefficients above 0.90, indicating strong agreement. As shown in **Figure 5**, parallelepiped performed poorly in delineating boll boundaries, resulting in markedly lower overall accuracies than mahalanobis distance and maximum likelihood (77.79% and 88.90%, respectively), together with substantially lower Kappa values (0.2808 and 0.7007). Differences in the underlying principles of these object-based algorithms led to distinct extraction outcomes. Overall, both mahalanobis distance and maximum likelihood provided reliable boll extraction, with overall accuracies above 96% and Kappa coefficients greater than 0.91.

**Table 4:** Accuracy assessment of cotton boll extraction based on object-based algorithms.

Object-based algorithms	In 2023		In 2024	
	Overall accuracy (%)	Kappa	Overall accuracy (%)	Kappa
Mahalanobis distance	96.6455	0.9147	97.0880	0.9305
Maximum likelihood	98.0130	0.9510	97.3139	0.9375
Parallelepiped	77.7879	0.2808	88.9042	0.7007

### 3.2 Analysis of boll-scale spectral features

#### 3.2.1 Pearson correlation between boll-scale spectral features and SBW

**Figure 6:**
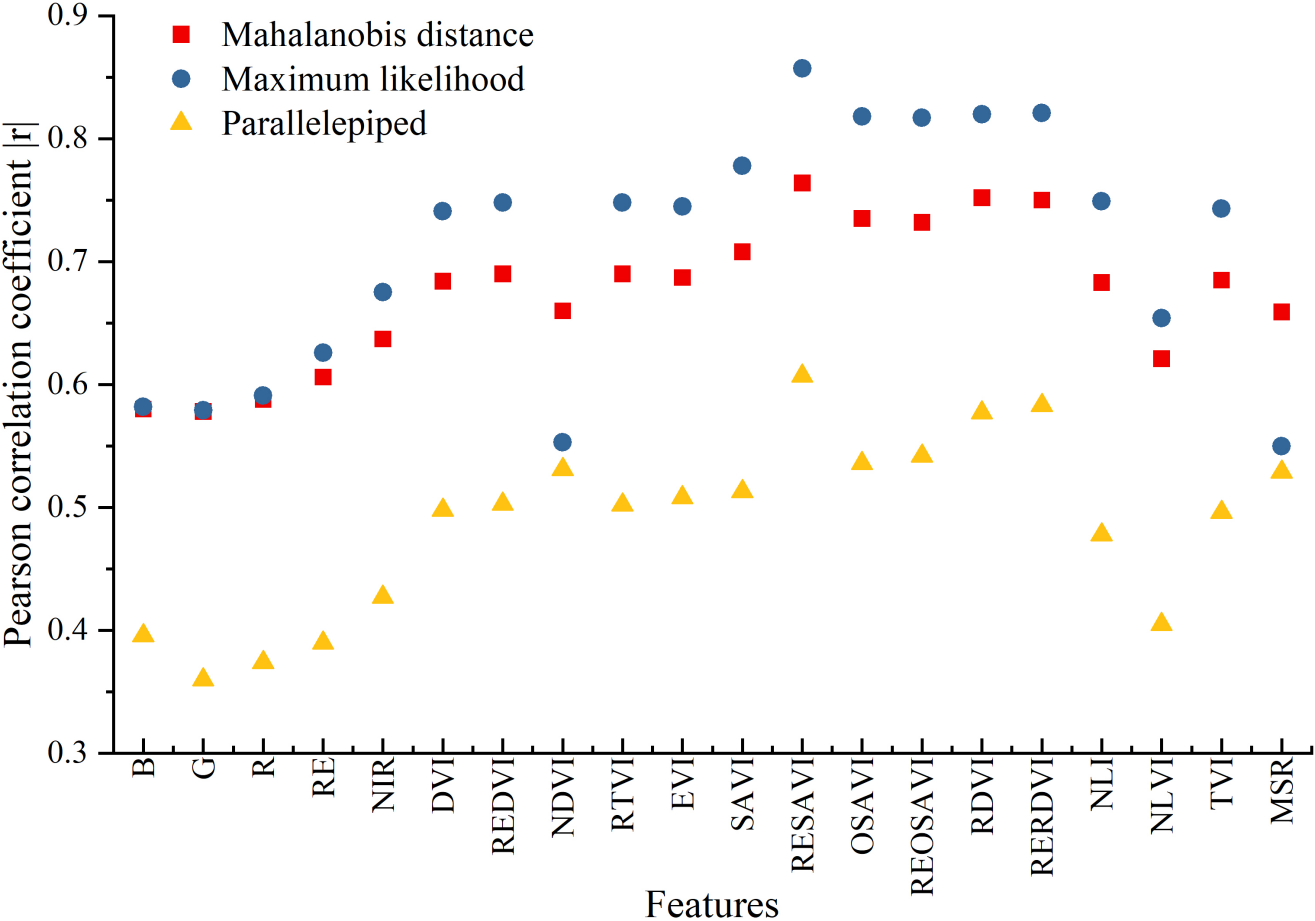
Pearson correlation analysis between boll-scale spectral features and SBW.

Cotton bolls were extracted using the three object-based algorithms (maximum likelihood, mahalanobis distance, and parallelepiped), and boll-scale spectral features were subsequently calculated. Pearson correlation analysis was then performed between these spectral features and SBW, with the results shown in **Figure 6**. Overall, the selected vegetation indices were positively correlated with SBW. The relative ranking of correlation coefficients between vegetation indices and SBW was generally consistent across indices derived from the three extraction algorithms. Notably, better boll extraction led to stronger correlations between extracted spectral features and SBW. Vegetation indices computed from bolls extracted by maximum likelihood exhibited the strongest correlations, followed by mahalanobis distance, whereas parallelepiped showed weaker relationships. Indices involving the near-infrared, red-edge, and red bands (e.g., RESAVI (RedEdge Soil-Adjusted Vegetation Index), RDVI (Renormalized Difference Vegetation Index), RERDVI (RedEdge Renormalized Difference Vegetation Index), OSAVI (Optimized Soil-Adjusted Vegetation Index), and REOSAVI (RedEdge-Optimized Soil-Adjusted Vegetation Index)) showed particularly strong correlations with SBW. Under the mahalanobis distance-, maximum likelihood-, and parallelepiped-based extraction results, the correlation coefficients of these indices with SBW exceeded 0.7, 0.8, and 0.5, respectively.

#### 3.2.2 SHAP analysis of boll-scale spectral features

**Figure 7:**
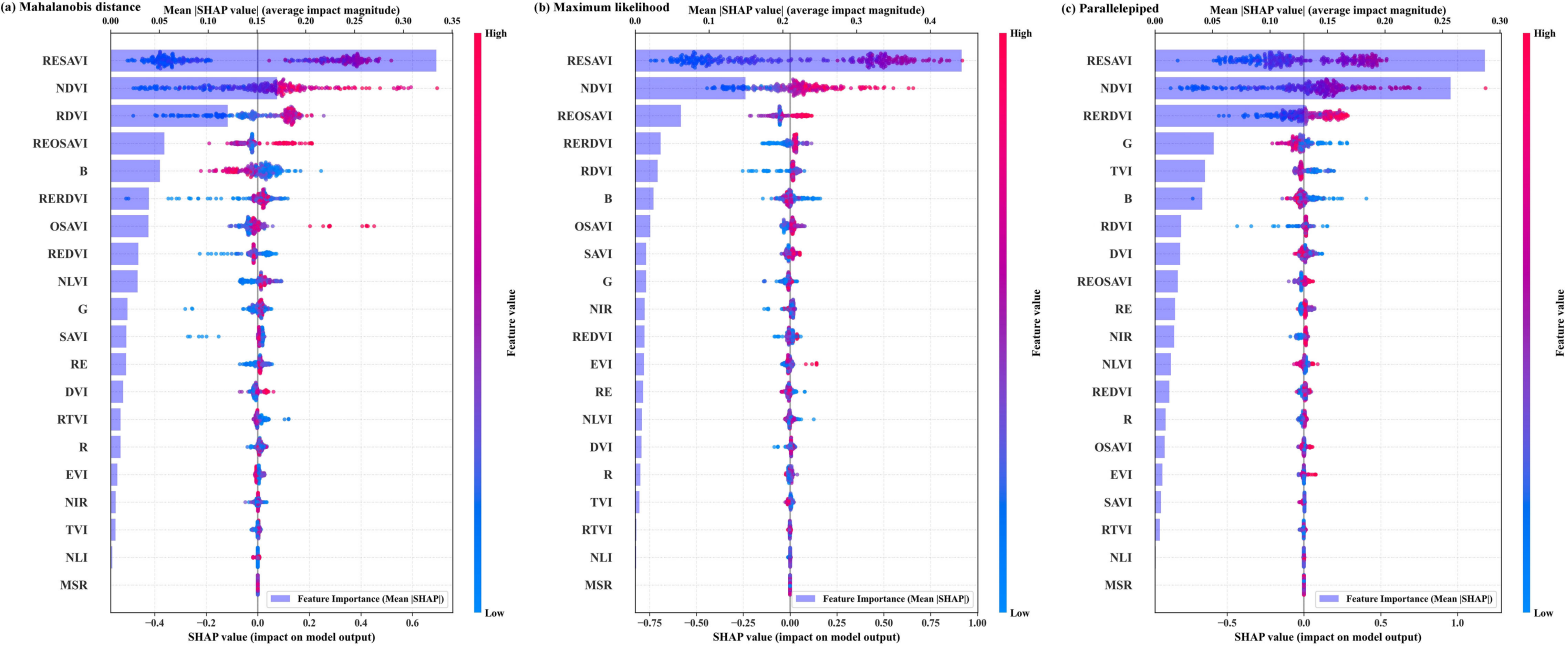
SHAP analysis of boll-scale spectral features: **(a)** Mahalanobis distance; **(b)** Mximum likelihood; **(c)** Prallelepiped.

To quantify the contribution of individual features to model predictions, SHAP analysis was performed on the spectral features derived from the boll-extracted imagery using an XGBoost model, and the results are visualized as SHAP summary plots in **Figure 7**. The absolute contribution of a given vegetation index varied slightly with the boll extraction results; however, the overall importance ranking remained largely consistent. Across all three boll extraction algorithms, the two most influential features were consistently RESAVI and NDVI (Normalized Difference Vegetation Index), both of which involve the near-infrared, red-edge, and red bands.

#### 3.2.3 Construction of the feature set for SBW estimation models

**Figure 8:**
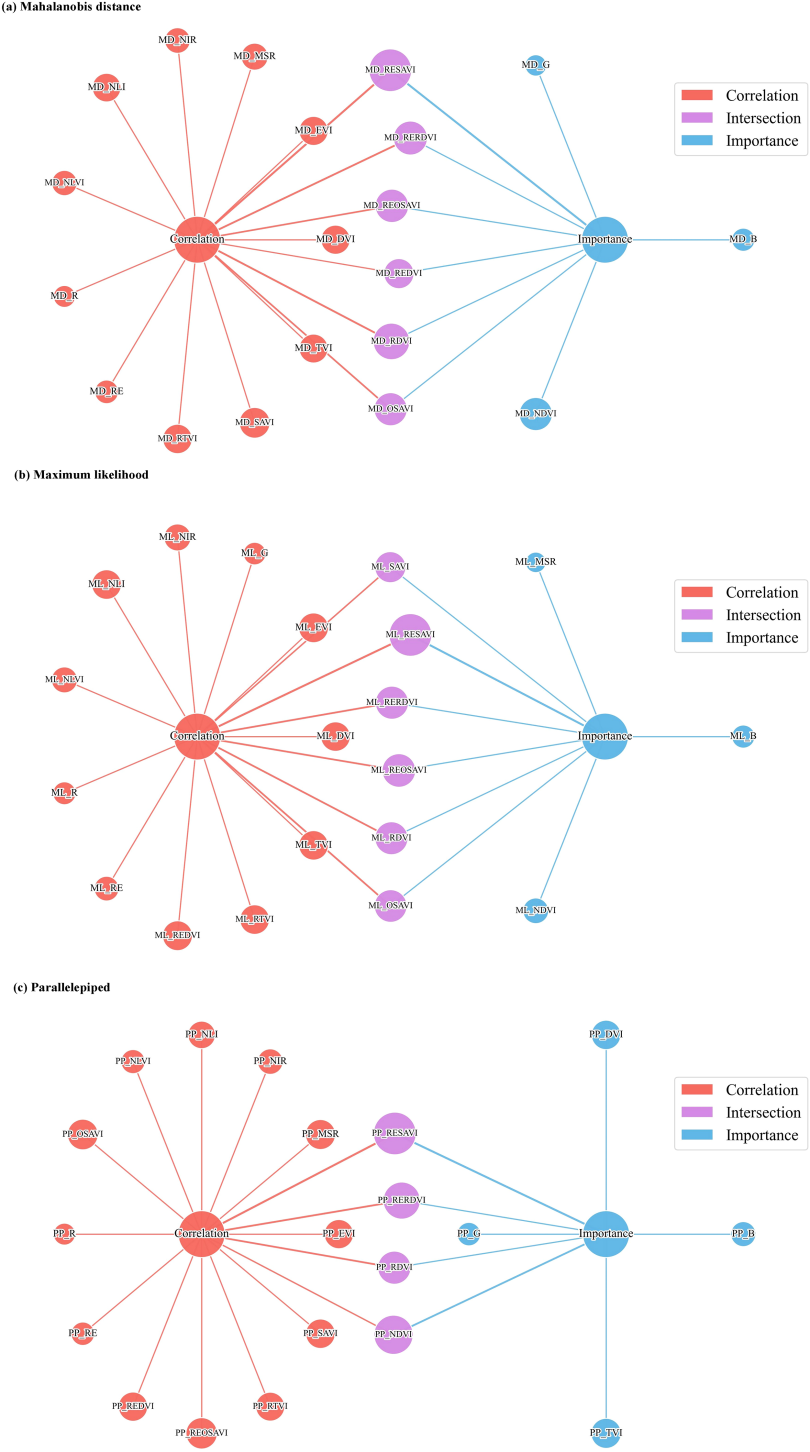
Feature fusion based on Pearson correlation and SHAP analysis: **(a)** Mahalanobis distance; **(b)** Maximum likelihood; **(c)** Parallelepiped.

A weighted averaging approach was used to integrate the Pearson correlation coefficients and SHAP importance values, and all vegetation indices were ranked accordingly. The SBW remote-sensing feature set was then constructed by selecting indices until the cumulative contribution reached 60%, with the results shown in **Figure 8**. Feature selection was influenced by the boll extraction algorithm. For mahalanobis distance, six indices were selected: RESAVI, RERDVI, REOSAVI, REDVI (RedEdge-Difference Vegetation Index), RDVI, and OSAVI. For maximum likelihood, six indices were selected: SAVI (Soil Adjusted Vegetation Index), RESAVI, RERDVI, REOSAVI, RDVI, and OSAVI. For parallelepiped, four indices were selected: RESAVI, RERDVI, RDVI, and NDVI.

### 3.3 Development of SBW estimation models based on fused features

**Figure 9:**
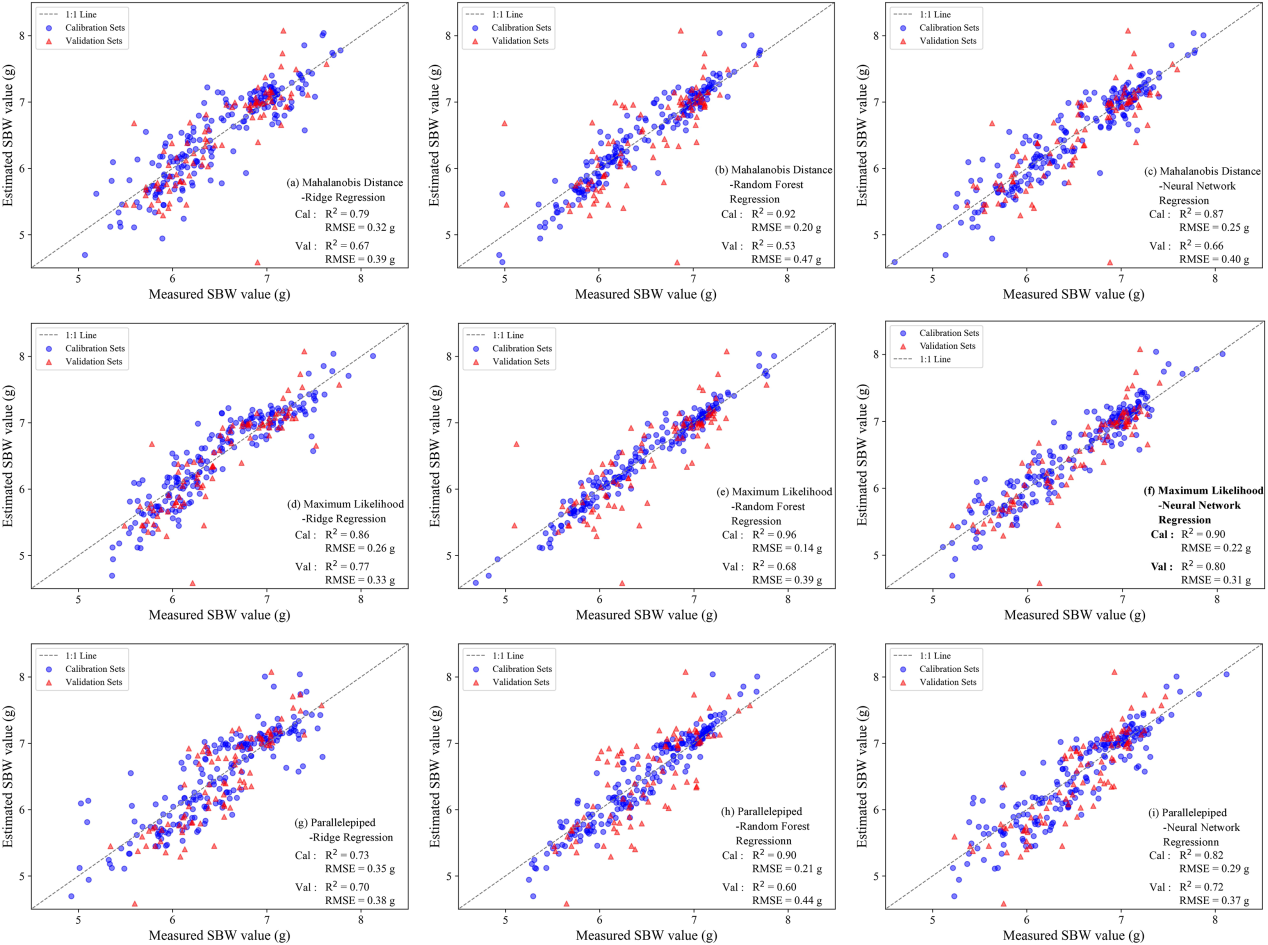
Accuracy of SBW estimation models based on fused features. **(a)** Mahalanobis Distance-Ridge Regression model; **(b)** Mahalanobis Distance-Random Forest Regression model; **(c)** Mahalanobis Distance-Neural Network Regression model; **(d)** Maximum Likelihood-Ridge Regression model; **(e)** Maximum Likelihood-Random Forest Regression model; **(f)** Maximum Likelihood-Neural Network Regression model; **(g)** Prallelepiped-Ridge Regression model; **(h)** Prallelepiped-Random Forest Regression model; **(i)** Prallelepiped-Neural Network Regression model.

SBW estimation models were developed using the selected features in combination with ridge regression, random forest regression, and neural network regression, and the results are shown in **Figure 9**. Models built on features derived from maximum likelihood-based boll extraction achieved higher accuracy than those based on mahalanobis distance and parallelepiped (R² = 0.68-0.80; RMSE = 0.39-0.31 g). Across the three regression approaches, on the calibration set, the nonlinear models (random forest regression, and neural network regression) yielded higher R^2^ values (0.90-0.96 and 0.82-0.90, respectively) than the linear ridge regression model (0.73-0.86). On the validation set, model performance decreased for all methods, yet the nonlinear models maintained their advantage. Considering the overall performance on both the calibration and validation sets, the SBW estimation model combining maximum likelihood-based boll extraction with neural network regression achieved the best performance, with a validation R^2^ of 0.80 and an RMSE of 0.31 g.

### 3.4 SBW spatial mapping and robustness validation

**Figure 10:**
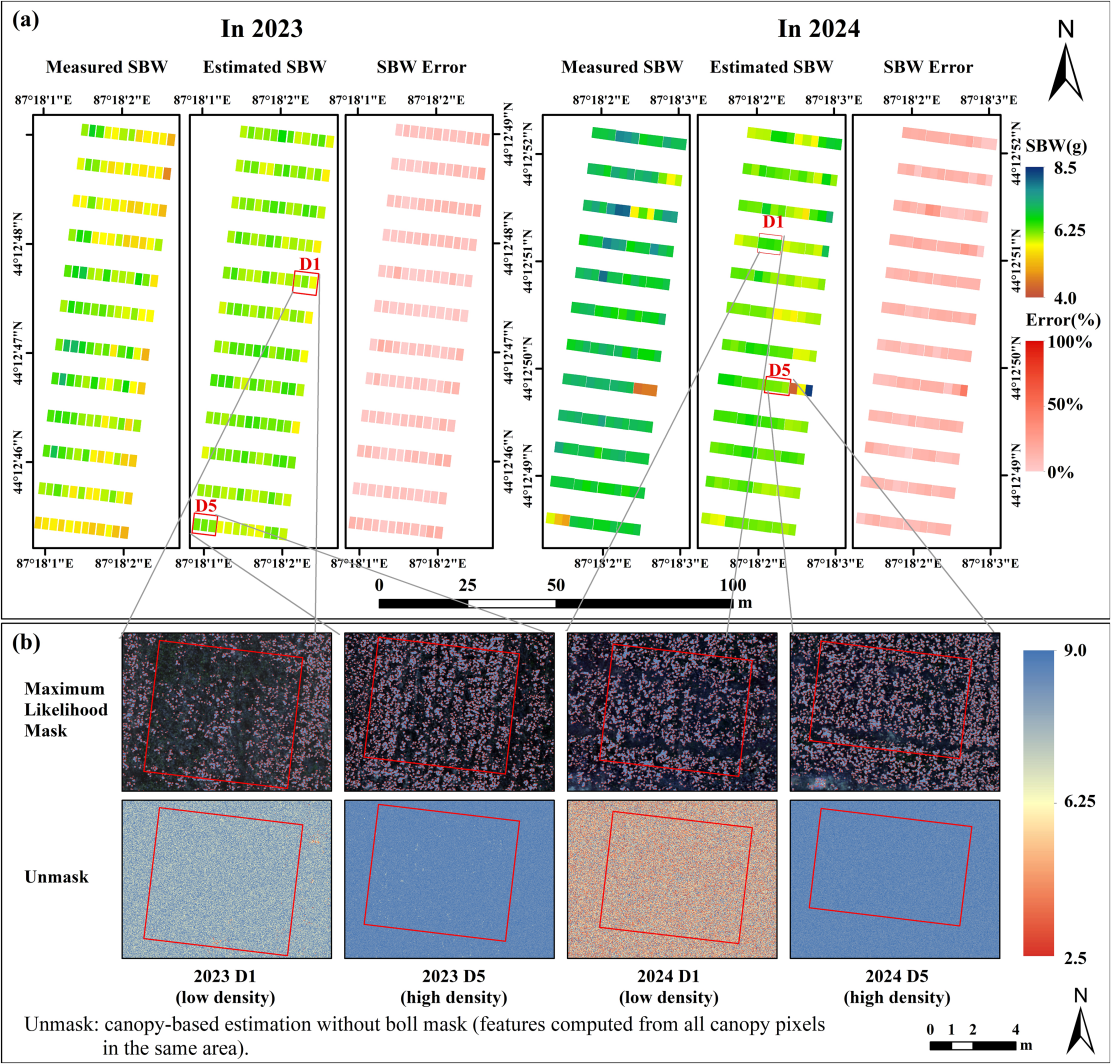
Plot-scale maps of cotton SBW derived from pixel-level predictions after boll masking, with comparisons to unmasked canopy-based estimation. **(a)** Spatial maps of measured cotton SBW, estimated cotton SBW, and estimation error for 2023 and 2024. **(b)** Zoomed-in examples for representative low-density (D1) and high-density (D5) plots. The upper row shows pixel-level predictions within the maximum likelihood mask, whereas the lower row shows unmasked canopy-based estimation (as indicated in the figure). The background is the red-green-blue composite, and colored pixels represent predicted cotton SBW. Pixel-level predictions were aggregated to plot-level values for breeding-oriented evaluation. Boll masking removed 74.72% of background pixels.

SBW was spatially mapped across the cotton field using the optimal modeling strategy that combines maximum likelihood-based boll masking with neural network regression, with the results shown in **Figure 10**. The results from both 2023 and 2024 indicate that this workflow can reliably estimate the spatial distribution of SBW, and predicted values were generally close to the measured values with relatively low errors (**Figure 10a**). In addition, the zoomed-in inset maps (**Figure 10b**) highlight pixel-level predictions for representative low- and high-density plots and provide a direct comparison between masked (object-based) and unmasked (canopy-based) estimation. Boll masking removed 74.72% of background pixels in the representative areas, demonstrating its ability to suppress background interference before aggregating pixel-level predictions to plot-level values for breeding evaluation.

**Figure 11:**
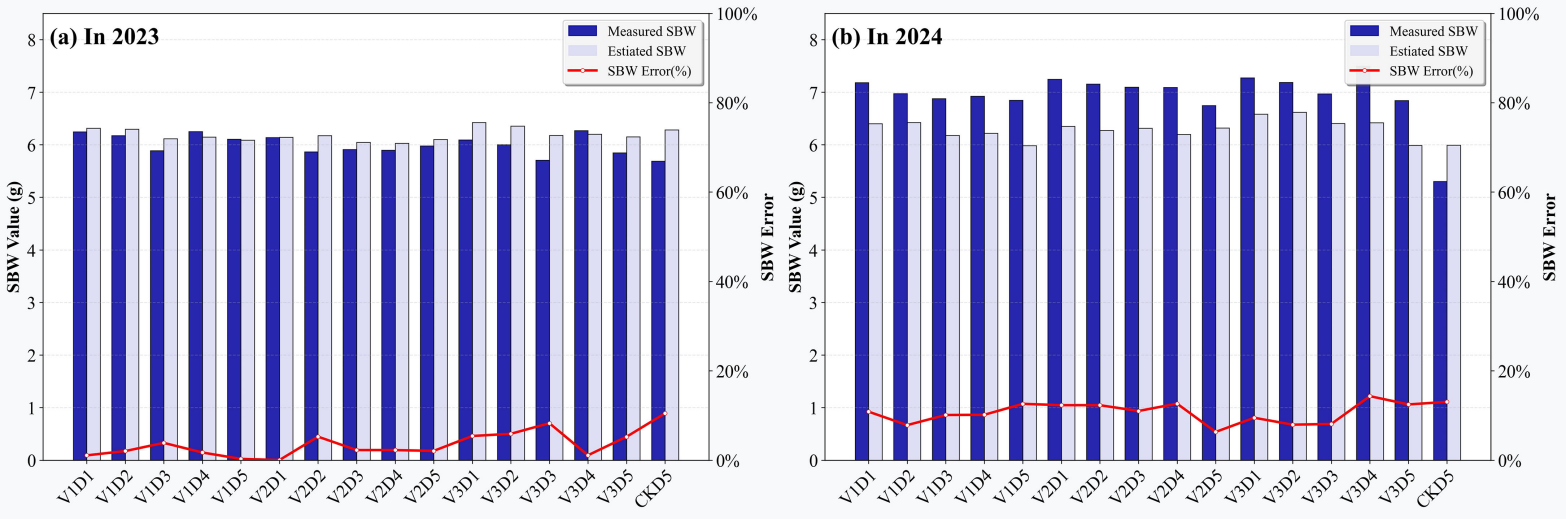
Robustness validation of the maximum likelihood-neural network regression-SBW model: **(a)** 2023 and **(b)** 2024.

The robustness of the maximum likelihood-neural network regression-SBW model was further confirmed by the results shown in **Figure 11**. Overall, the model exhibited strong robustness, with estimated values closely matching the measured values. A slight overestimation was observed in the low-value range, whereas a slight underestimation occurred in the high-value range. Overall, the maximum likelihood-neural network regression-SBW model achieved accurate SBW estimation. Notably, the relative errors across different combinations of years, varieties, and planting densities were all below 15%, further demonstrating the robustness of the maximum likelihood-neural network regression-SBW model.

## 4 Discussion

### 4.1 Role of object-based boll extraction in subsequent retrieval

Object-based approaches are well suited for delineating morphologically complex, small-scale targets in high-resolution imagery (Blaschke, 2010). Consistent with this, our results showed that all three object-based supervised classification algorithms effectively separated cotton bolls from background components. Among them, maximum likelihood achieved the highest overall accuracy (≥96.6455%) and Kappa coefficient (≥0.9147) across the two growing seasons. The Mahalanobis distance classifier demonstrated higher sensitivity to the distribution and quantity of the training samples provided. In contrast, the parallelepiped classifier, due to its reliance on simple, axis-aligned decision rules, proved particularly prone to misclassifying pixels in spectrally ambiguous zones such as boll boundaries and shaded areas.

Importantly, the quality of segmentation is not only reflected by classification accuracy metrics but also by the strength of subsequent spectral-trait relationships. Vegetation indices extracted using the maximum likelihood-based mask generally exhibited higher correlations with SBW and higher model importance than those derived from the other two algorithms. This indicates that high-quality boll extraction can effectively improve the “purity” of spectral signals by reducing interference from soil and residual leaves, thereby providing more reliable inputs for SBW retrieval. Previous studies on deep-learning-based boll detection and boll-index construction have likewise shown that extraction quality is a prerequisite for accurate yield estimation (Sun et al., 2020; Li et al., 2022; Reddy et al., 2024), and our findings provide further evidence for this conclusion from the perspective of object-based classification.

### 4.2 Response mechanisms between boll-scale spectral features and SBW

Building on boll extraction, this study identified SAVI, OSAVI and their red-edge variants, as well as RDVI and RERDVI, as core predictors for SBW estimation from multiple categories of vegetation indices. These indices share two key characteristics: (i) they are constructed using the red, red-edge, and near-infrared bands, and (ii) they generally incorporate soil-adjustment terms or re-normalized formulations. This is consistent with the spectral environment of cotton fields after boll opening. On the one hand, cotton lint exhibits high brightness in the visible bands, and some wavelengths are easily confounded with exposed soil; therefore, “greenness-type” indices such as NDVI alone are insufficient to characterize cotton bolls effectively. On the other hand, defoliation increases canopy porosity and the proportion of exposed soil, thereby amplifying the influence of soil brightness variability on spectral signals. Soil-adjusted vegetation indices mitigate this effect by introducing correction factors, whereas red-edge-related indices are more sensitive to residual green tissues and structural changes around the fiber (Huete, 1988; Rondeaux et al., 1996).

In this study, the Pearson correlations analysis and SHAP-based importance rankings were highly consistent, suggesting that vegetation indices incorporating red-edge information and designed to reduce soil background effects are not only strongly associated with SBW in univariate analyses but also contribute robustly in multivariate models. Compared with studies that estimate cotton yield or SBW from canopy-averaged spectra (Ma et al., 2022), our approach derives spectral predictors from boll pixels identified by object-based extraction. This design provides a closer correspondence between remote-sensing signals and yield components and supports a more physically interpretable relationship between spectral features and SBW.

### 4.3 Performance, error structure, and application potential of SBW estimation models

Using the selected features, we further evaluated the potential of different machine-learning algorithms for SBW estimation. Overall, nonlinear models outperformed the linear model, indicating a certain degree of nonlinearity between boll-scale spectral signals and SBW, which is consistent with previous findings on estimating cotton and other crop yields using UAV imagery and machine learning (Ashapure et al., 2020). Among the tested approaches, the neural network model built on maximum likelihood-based boll extraction achieved a favorable balance between the calibration and validation sets, with a validation R^2^ of 0.80 and an RMSE of 0.31 g. Moreover, the relative errors remained below 15% across different years, varieties, and planting densities, demonstrating good robustness under the experimental conditions.

Notably, error analysis revealed a systematic pattern: slight overestimation at low SBW values and some underestimation at high SBW values. This pattern is more than a simple “regression-to-the-mean” statistic; it reflects fundamental constraints in spectral-trait modeling. We attribute this non-linear bias to two intertwined factors. First, at the high-SBW end, the observed underestimation is likely driven by spectral saturation effects, where the sensitivity of broadband vegetation indices diminishes as biomass or yield increases, compressing the dynamic range of the spectral response (Ashapure et al., 2020). Concurrently, occlusion and shading within dense canopies may exacerbate signal saturation. Second, at the low-SBW end, the overestimation may stem from increased interference from background soil and senescent material in mixed pixels, coupled with a potential data imbalance where extreme low-value samples are underrepresented in the training set. Together, these physical and statistical factors cause the model’s predictions to be biased toward the mean, particularly at the trait distribution tails. Future research may address this issue by employing less-saturating hyperspectral or red-edge indices, extending the saturation point through fusion of multimodal data, and implementing strategic oversampling for extreme phenotypes.

From an application perspective, SBW spatial maps can be used together with boll-number or yield maps. In breeding trials, such joint interpretation can help distinguish genotypes characterized by “many bolls with low SBW” from those with “few bolls but high SBW.” In production management, SBW maps can provide complementary information for optimizing planting density, guiding defoliant application, and determining harvest timing, thereby extending existing yield-estimation frameworks that rely on canopy height or fractional cover (Niu et al., 2023; Huang et al., 2025).

### 4.4 Limitations and future perspectives

It should be noted that this study has several limitations. First, the experiment was conducted at a single site in northern Xinjiang, where soil type and management practices were relatively consistent. Although multiple varieties and planting densities were included, the transferability of the proposed model across different agro-ecological zones and cultivation systems remains to be validated. Yield estimation models are often affected by factors such as variety type, mulching practices, and management level when applied across regions (Niu et al., 2023; Ma et al., 2022). Second, the current object-based segmentation relies on manually labeled training samples and is also dependent on image spatial resolution and radiometric calibration quality. To scale up to large production fields, it may be necessary to adopt deep-learning frameworks such as instance segmentation to reduce annotation costs and improve adaptability under complex backgrounds and small-target scenarios (Li et al., 2022; Reddy et al., 2024). In addition, the regression models in this study were built solely on spectral features, without integrating structural information derived from digital elevation models or 3D point clouds, or agronomic covariates such as meteorological and soil variables. Multi-source data fusion has been shown to improve the robustness and cross-year generalization of crop yield estimation models (Ashapure et al., 2020).

Therefore, future work should pursue two complementary directions to enhance model generalizability. First, expanding sampling campaigns across multiple ecological regions is essential to build a more representative and diversified training dataset. Second, on the methodological front, it is crucial to explore techniques such as transfer learning or domain adaptation. These approaches could potentially leverage the model knowledge gained from data-rich source environments (like the current study site) to improve performance in new target regions with limited ground truth data, thereby reducing the dependency on large-scale labeled samples from every new zone. Ultimately, integrating these efforts within a framework of “high-quality segmentation + multi-source feature fusion” will support the joint retrieval of SBW and other yield-component traits. This will provide more comprehensive phenotypic information to guide precision cotton production and breeding decisions.

## 5 Conclusion

This study developed an SBW estimation pipeline that integrates object-based cotton boll segmentation, vegetation indices designed to reduce soil background effects and incorporating red-edge information, and machine-learning regression. The pipeline was evaluated using data from a two-year field experiment conducted in a typical drip-irrigated cotton system in Xinjiang, China, in which four varieties were tested under five planting-density treatments. The results demonstrate that maximum likelihood segmentation produces reliable boll masks and substantially strengthens the association between boll-scale spectral signals and SBW. Vegetation indices derived from the red-edge bands and formulated to account for soil background, including the SAVI, OSAVI, and their red-edge variants, were identified as key predictors for SBW estimation. Based on these features, the model that combines maximum likelihood-based boll extraction with neural network regression achieved good accuracy and robustness across years, varieties, and planting-density. Overall, our findings verify the feasibility of organ-scale SBW estimation from UAV multispectral imagery and provide methodological support for incorporating yield-component traits into high-throughput phenotyping and precision cotton production.

## Data Availability

The raw data supporting the conclusions of this article will be made available by the authors, without undue reservation.
